# Overcoming Aminoglycoside Enzymatic Resistance: Design of Novel Antibiotics and Inhibitors

**DOI:** 10.3390/molecules23020284

**Published:** 2018-01-30

**Authors:** Sandra G. Zárate, M. Luisa De la Cruz Claure, Raúl Benito-Arenas, Julia Revuelta, Andrés G. Santana, Agatha Bastida

**Affiliations:** 1 Facultad de Tecnología-Carrera de Ingeniería Química, Universidad Mayor Real y Pontificia de San Francisco Xavier de Chuquisaca, Regimiento Campos 180, Casilla 60-B, Sucre, Bolivia; zarate.sandra@usfx.bo; 2 Facultad de Ciencias Químico Farmacéuticas y Bioquímicas, Universidad Mayor Real y Pontificia de San Francisco Xavier de Chuquisaca, Dalence 51, Casilla 497, Sucre, Bolivia; mariafeliz3@hotmail.com; 3 Departmento de Química Bio-Orgánica, Instituto de Química Orgánica General (CSIC), Juan de la Cierva 3, 28006 Madrid, Spain; rbenito@iqog.csic.es (R.B.-A.); julia.revuelta@iqog.csic (J.R.)

**Keywords:** antibiotic resistance, combination therapy, bi-substrate inhibitors, decoy acceptors

## Abstract

Resistance to aminoglycoside antibiotics has had a profound impact on clinical practice. Despite their powerful bactericidal activity, aminoglycosides were one of the first groups of antibiotics to meet the challenge of resistance. The most prevalent source of clinically relevant resistance against these therapeutics is conferred by the enzymatic modification of the antibiotic. Therefore, a deeper knowledge of the aminoglycoside-modifying enzymes and their interactions with the antibiotics and solvent is of paramount importance in order to facilitate the design of more effective and potent inhibitors and/or novel semisynthetic aminoglycosides that are not susceptible to modifying enzymes.

## 1. Introduction

The continuous appearance of bacterial strains resistant to most antibiotics already known, along with the scarce perspective of new bactericidal agents coming out in the near future, have placed bacterial multi-drug resistance (MDR) among the most pressing issues for worldwide health [1,2,3]. Furthermore, the increasing number of hospital MDR infections has boosted the interest for new aminoglycoside antibiotics for clinical use, and consequently research on this topic has drawn renewed attention. Aminoglycosides are a group of natural antibiotics from *Streptomyces* that have been used in clinical practice for more than 50 years [4]. Their potent bactericidal activity relies upon binding specifically to the 16S rRNA of the 30S ribosomal subunit, thus interfering with protein synthesis [5,6]. However, the first resistant bacterial strains began to appear in the 1960s due to a high rate of dissemination via R-plasmids, transposons, and integrons [7,8]. In an attempt to overcome this emerging resistance to natural antibiotics, the first semi-synthetic aminoglycosides, such as amikacin, dibekacin, isepamicin, and netilmicin, were introduced during the 1970s [9,10]. The discovery of gentamicins, a newer family of aminoglycosides isolated from *Micromonospora*, contributed to a come-back of this class of bactericidal agents in clinical practice [11]. These antibiotics were very active against *Pseudomonas aeruginosa*, a tougher micro-organism less susceptible to the original aminoglycosides, which unfortunately soon after developed resistance through the ANT(2″) enzyme [12,13]. Butirosine, another aminoglycoside discovered later, was able to avoid inactivation by APH(3′) and ANT(2″) enzymes [14]. There is a large number of aminoglycoside antibiotics, but the resistance mechanisms developed by micro-organisms increase with the frequency of their use.

## 2. Understanding the Modifying Enzymes

**Figure 1:**
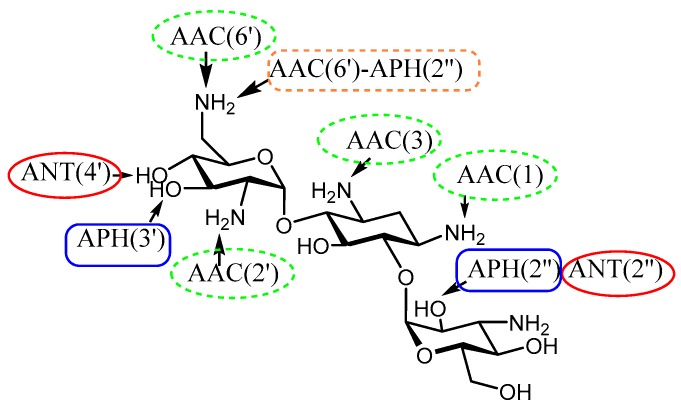
Susceptible positions in Kan B to AME modification.

Tolerance towards antibiotics can be achieved through different mechanisms. However, the most prevalent in the clinic is that due to enzymatic modification that renders aminoglycosides of decreased affinity for their natural primary target, 16S rRNA [15,16,17]. There are three families of aminoglycoside-modifying enzymes (AMEs): *N*-acetyltransferases (AACs) that acetylate an amino group using acetyl-Coenzyme A; *O*-nucleotidyltransferases (ANTs) that transfer an adenyl group from ATP to a hydroxyl group of the antibiotic; and *O*-phosphotransferases (APHs), which phosphorylate a hydroxyl group also employing ATP (Figure 1). The emergence of bifunctional enzymes (i.e., APH(2″)-AAC(6′)) that are able to modify almost all aminoglycoside antibiotics presents a huge challenge that new aminoglycosides will have to overcome [18] (Figure 1).

AACs are found both in Gram-positive and Gram-negative bacteria and are able to reduce the affinity of the antibiotic for the receptor 16S rRNA by 4 orders of magnitude (Table 1). This family of enzymes is the largest within the AMEs, with 48 sequences identified so far, and they all weigh around 20–25 kDa and comprise a wide range of positions susceptible to modification (6′, 2′, *N*-1, and *N*-3). Mutagenesis studies have shown that just the mutation of a single amino acid of the AAC can modulate the specificity for the antibiotic. For example, AAC(6′)-I and AAC(6′)-II share the capacity to modify kanamycin but they differ in their propensity to acetylate amikacin or gentamicin C. The structural gene of the AAC(3) and AAC(6′) is generally found on transposable elements [19]. AAC(3) enzymes were the first to be described to confer resistance to gentamicin (G), kanamycin (K), fortimicin (F), and tobramycin (T) [20] and can acetylate either *N*/*O* groups of the aminoglycoside. Five AAC(2′) enzymes are encoded in Mycobacteria and one has been crystalized from *Mycobacterium tuberculosis.* AAC(2′) from *M. tuberculosis* catalytically and enthalpically favors those aminoglycosides with amine/hydroxyl groups at the 2′ position and shows an increase in affinity for all antibiotics when CoA is present. This AME exhibits a high tolerance towards the antibiotic molecule (4,5- and 4,6-substitution), and it is thought to be modulated by water molecules that mediate between aminoglycoside and side chain carboxylate groups of the receptor. Moreover, side chain reorientation relative to the apo-enzyme upon binding of the substrates also contributes to the plasticity of this enzyme [21]. AAC(6′) enzymes are the most prevalent in clinical strains, conferring resistance to amikacin (A), gentamicin (G), kanamycin (K), neomycin (N), dibekacin (D), sisomicin (S), isepamicin (I), and tobramycin (T) and has an ordered kinetic mechanism. Three-dimensional (3D) structures of AAC(6′), AAC(3), and AAC(2′) have been described with a remarkable conservation of the overall structure, albeit with low amino acid sequence identity [22,23].

APHs are the second-most abundant family of AMEs and confer resistance to aminoglycosides in *Enterococcus* and *Staphylococcus* strains (Table 2). There are seven types of APHs with 30 kDa mass and high sequence homology (20–40%) in their C-terminal end [24]. APH(3′)-IIIa is usually used as a resistance marker and its 3D structure has been described several times [25,26]. APH(3′)-IIIa promiscuity seems to be governed by disordered elements that adopt well-defined conformations when the aminoglycoside is bound [27]. APH(3′)-IIa from *Enterococcus faecalis* is able to phosphorylate the 3′ and 5′-OH groups, giving rise to di-phosphorylated aminoglycosides [28] through an ordered sequential mechanism in which the binding of ATP is followed by the antibiotic [29]. An interesting property of this enzyme is that it is competitively inhibited by tobramycin, which one would expect not to be a substrate because it lacks a free 3′-hydroxylgroup. APH(2″) is a very promiscuous enzyme based on the range of susceptible positions in the antibiotic skeleton (2″, 3′, 3″, and 5″); it represents an important resistance element in Gram-positive bacteria, and this enzyme uses Guanosine Triphosphate (GTP) as the most efficient donor substrate over ATP. Oddly enough, many other APHs have been identified, such as APH(6), APH(3″), APH(9), APH(4), and APH(7), but their occurrence is strikingly uncommon in the clinic. APH(9)-Ia exhibits a similar folding to that of the APH(3′) and APH(2″) enzymes, but it differs significantly in its substrate binding area and in the fact that it undergoes a conformational change upon ligand binding.

ANTs are the smallest family of AMEs, with four crystalized structures, ANT(2″), ANT(3″), ANT(4′), and ANT(6′), as can be seen in the following table (Table 3) [30,31,32,33,34,35].

Genes coding for ANT enzymes are found in plasmids, transposons, and chromosomes. ANT(2″)-Ia is able to modify gentamicin, tobramycin, dibekacin, sisomicin, and kanamycin [30], while ANT(4′)-Ia is active against almost all aminoglycosides and other aminoglycosides with 4′/4″-OH groups [33]; ANT(3″) inactivates streptomicyn and spectinomycin [32], while ANT(6) and ANT(9) can modify only streptomycin and spectomycin, respectively [35,36]. ANT(2″)-Ia and ANT(4′)-Ia are clinically relevant proteins and only share 27% amino acid homology. ANT(4′)-Ia is a dimeric enzyme with two active sites, and recognizes all nucleotides triphosphate and almost all 4,5 or 4,6-aminoglycosides except those from the streptomycin family [37,38]. The 3D structure of ANT(4′)-Ia was the first one to be described, and its complex with kanamycin revealed that several active site residues interact via hydrogen bonds with the glucosamine and 2-deoxystreptamine moiety, but relatively fewer residues interact with the 3′-aminoglucose sugar, this being important knowledge for the design of antibiotics or inhibitors. The antibiotic binding pocket is covered by negatively charged residues, where Glutamic 145 acts as a general base for the activation of the 4′-OH group of kanamycin, thus preparing the antibiotic for the attack of the Mg-ATP stabilized by lysine 149, which facilitates the nucleophilic attack. Overall, the dominant role of electrostatics in aminoglycoside recognition, in combination with the enzyme anionic regions, confers to the protein/antibiotic complex a highly dynamic character. The kinetic mechanism is an ordered process, where the antibiotic binds first to the active site and later on to the Mg-ATP. Determination of the order of product release revealed that PPi is discharged first, followed by the AMP-aminoglycoside, all in agreement with an ordered Bi-Bi mechanism [39]. ANT(2″)-Ia from *Klebsiella pneumoniae* has an ordered sequential mechanism, where the presence of the two substrates at the same time is essential (Mg-ATP binds before the aminoglycoside) [40,41,42,43]. The observed promiscuity of ANT(2″)-Ia from *Pseudomonas aeruginosa* is not only due to binding cleft size, but also it can be controlled by ligand modulation on dynamic, disordered, and thermodynamic properties of ANT under cellular conditions [44]. ANT(6′), a 37 kDa protein, transfers an adenyl group from ATP to the 6′-hydroxy function on the aminoglycoside, thus leading to a sharp decrease in the drug affinity for its target RNA. The conformational behavior of streptomycin, both in the free and the protein-bound states, was studied by NMR experiments given that the 3D structure is in a free form [45,46]. The streptomycin is characterized by a high degree of flexibility in solution, but this equilibrium is clearly altered upon binding to the enzyme. ANT(6′) showed a clear specificity for nucleotides that incorporate a purine ring, and regarding the aminoglycoside counterpart, it is very specific: it only recognizes streptomycin. In contrast, the 3D structure and the kinetic mechanism of ANT(9) has not been resolved so far.

It was in 1986 when the first bifunctional enzyme was described, AAC(6′)-Ie-APH(2″)-Ia from *Enterococcus faecalis* [47]; a structure-function analysis with various aminoglycosidic substrates revealed an enzyme with a broad specificity in both enzymatic activities catalyzing *N*- and *O*-acetylation. The AAC(6′)-APH(2″) from *Staphylococcus aureus* enzyme confers resistance to gentamicin, kanamycin, tobramycin, and, when overexpressed, to amikacin, presenting a dramatic negative impact on clinical therapy [48]. Later on, other bifunctional enzymes were also described: ANT(3″)-Ii-AAC(6′)-IId from *Serratia marcescens* [49] and AAC(3)-Ib-AAC(6′)-Ib from *Pseudomonas aeruginosa* [50] (Table 4). 

The adenyltransferase domain of ANT(3″)-Ii-AAC(6′)-IId appears to be highly specific for the aminoglycoside, while the acetyltransferase domain shows a broad substrate tolerance [51]. Kinetic analysis of the mechanism of ANT(3″)-Ii points towards a Theorell–Chance type of reaction, with ATP binding to the active site before the aminoglycoside, and once the reaction has occurred, the product is the last to be released. However, the AAC(6′)-IId domain follows an ordered Bi-Bi mechanism in which the antibiotic is the first to bind in the active site, and CoA is released prior to the modified aminoglycoside. Also, structural studies have revealed that this enzyme contains dynamic segments that modulate before and after aminoglycoside binding. In the case of AAC(3)-Ib-AAC(6′)-Ib, both domains follow a sequential ordered kinetic mechanism in which the acetyl-CoA binds first to the active site, followed by the aminoglycoside, and the CoA is the last product to be released [52]. Despite this interesting behavior, to date only the crystal structure of some APH(2″) domains have been described.

## 3. Semi-Synthetic Aminoglycoside Derivatives

**Figure 2:**
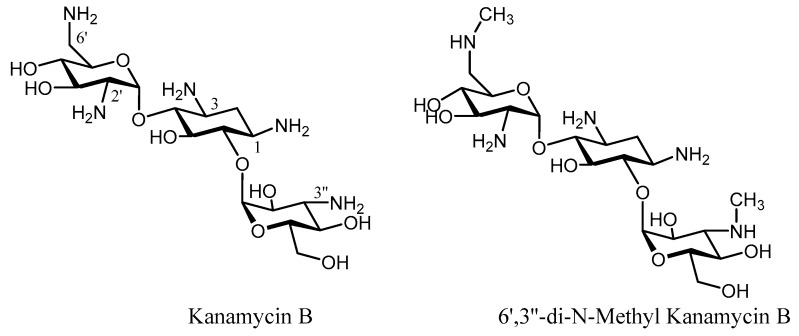
Structures of Kanamycin B and 6’,3’’-di-N-Methyl Kanamycin B.

The emergence of resistance towards the first generation of aminoglycosides led to increased efforts to identify similar antibiotics that were not susceptible to resistance [53,54,55,56,57,58]. From a conceptual point of view, one of the most simple and straightforward strategies to generate new derivatives that are not susceptible to enzymatic inactivation relies on the modification or elimination of those functional groups (OH/NH_2_) that can be altered by the enzyme. However, this should only be taken into consideration when such functional groups are not involved in key contacts with the receptor (16S rRNA) so that their biological activity is maintained. Tobramycin (3′-deoxy-kanamycin B) showed high activity against strains expressing APH(3′), and it is a good competitive inhibitor of this enzyme [59]. Dibekacin (3′,4′-dideoxy-kanamycin B) was the first rationally designed semi-synthetic aminoglycoside based on the 3′ phosphorylation of kanamycin B, being effective against *Staphylococcus* and *Pseudomonas*, but unfortunately still susceptible to ANT(2″) [60] and the bifunctional enzyme AAC(6′)-APH(2″) [61]. In contrast, fewer examples of deoxygenation on neomycin-related antibiotics have been reported [62]. The number and positions of amino groups play a significant role in the aminoglycoside activity. Methylation of the *N*-6′ and *N*-3″ positions in kanamycin B yielded a compound (6′,3″-di-*N*-methyl-kanamycin B) that displayed activity against resistant strains, but showed a weaker bactericidal activity than its natural antibiotic [63] (Figure 2).

**Figure 3:**
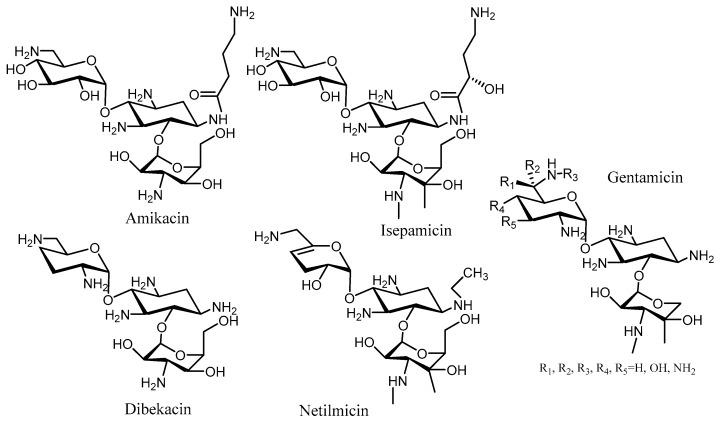
Structure of kanamycin class of aminoglycosides.

The observation that butirosin, a *N*-1 derivative of the 2-deoxystreptamine moiety, is poorly modified by APH(3’), prompted the synthesis of several kanamycin and neomycin derivatives bearing an *N*-1 modification. Kanamycin derivatization at this position with a (*S*)-4-amino-2-hydroxybutyryl (AHB) group gave rise to amikacin, which has proven to be a very effective aminoglycoside antibiotic in clinic [64] (Figure 3). This compound was able to arrest the cell growth of strains expressing the AAC(1), APH(3′)-Ia, and ANT(2′) enzymes [65]. The *N*-1 modification of dibekacin with an AHB group provided arbekacin, which was used in clinic against *Pseudomonas* and *Staphylococcus* [66] (Figure 3). Arbekacin is a substrate of the bifunctional enzyme AAC(6′)-APH(2″), but cannot be modified by the APH(3′) or ANT(4′) present in some Methicillin-resistant *Staphylococcus aureus* (MRSA) strains [67,68]. The trend of antibacterial activity of these aminoglycoside derivatives with an *N*-1 AHB group is similar to a 3′-deoxygenation in the antibiotic skeleton. Some other functionalities at the *N*-1 position were also studied, but were much less active against *P. aeruginosa.*

**Figure 4:**
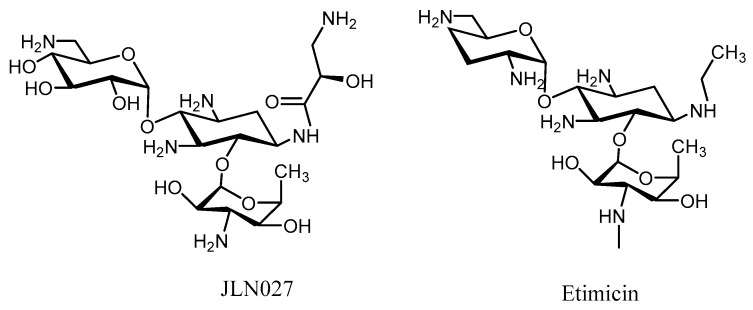
Aminoglycosides with *N*-1 kanamycin derivatives.

Further elaboration upon these *N*-1 derivatives yielded two new derivatives, JLN027 and etimicin, that are more effective than gentamicin, amikacin, and tobramycicn [69,70,71], but still have encountered resistance to some MRSA strains [72,73,74,75] (Figure 4).

**Figure 5:**
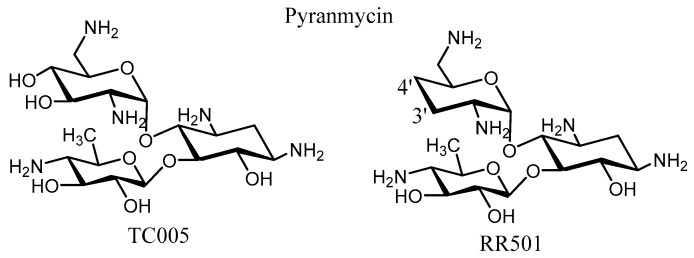
Structure of kanamycin B analogs (Pyranmycin).

Modification of the *N*-6′, *N*-2′, *N*-3, and *N*-1 positions through the synthesis of deaminated aminoglycosides (neamine or kanamycin B) has shown a dramatic loss of enzymatic susceptibility towards APH(3′)-Ia/IIa while still retaining the antibacterial activity, probably due to a decreased overall positive charge on the antibiotic. AMEs do not show activity against pyranmycins, which are a group of semisynthetic derivatives of aminoglycosides that differ from regular aminoglycosides in that they contain a pyranose in place of a furanose at the *O*-5 position of neamine, giving rise to a derivative with low cytotoxicity (TC005 derivative) [76] (Figure 5). In order to improve the derivatives, Chang’s group prepared some 3′,4′-dideoxygenated pyranmycin and kanamycin derivatives giving resistance to AME (RR501) [77,78] (Figure 5). These simplified skeletons proved highly effective against several pathogenic bacterial strains, such as *P. aeruginosa* and *S. aureus*.

**Figure 6:**
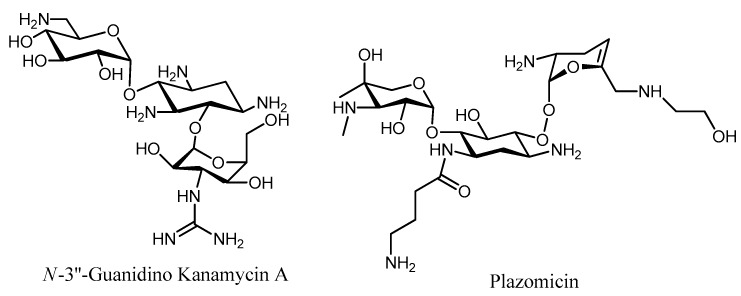
Structure of the *N*-3’’-Guanidino Kanamycin A and Plazomicin.

Selective modification of the *N*-3″ amino of kanamycin A into a guanidine group gave *N*-3″-guanidino kanamycin A (Figure 6), which presented protection against inactivation performed by ANT(4′), APH(3′), and AAC(6′), while maintaining its antibiotic activity [79]. The protecting effect of the guanidine group at the 3″-position was rationalized in terms of a binding hindrance with the respective inactivating enzymes, which presumably does not take place within the A-site.

Plazomicin (PLZ, formerly ACHN-490) represents another example of amino modification, where the *N*-6’, *N*-1, and *N*-3″ positions have been substituted with different ramifications (Figure 6). This aminoglycoside is not affected by any known AME except for AAC(2′), making it one of the few examples that is currently in clinical use, retaining activity against most of the clinical isolates (Minimum Inhibitory Concentration, MIC 4 μg/mL) [80,81,82]. These new aminoglycosides constitute our most promising defense alternative for the treatment of resistant MRSA strains. PLZ is currently in Phase 3 clinical trials for patients with bloodstream infections or nosocomial pneumonia and it is an important new weapon in the pipeline to fight antibiotic resistance.

**Figure 7:**
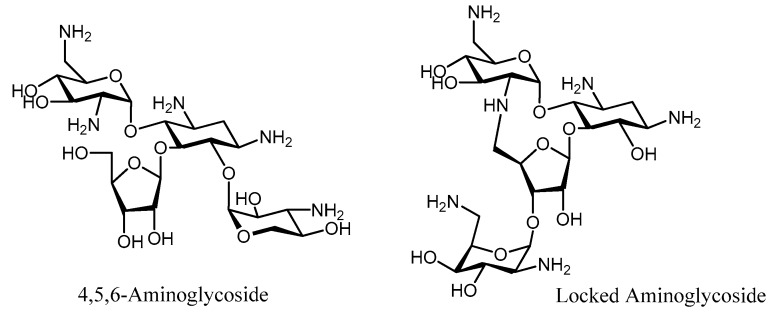
Structure of the 4,5,6-Neomycin derivative and constrained neomycin.

The aminosugar ring in aminoglycosides provides these molecules with high affinity for the prokaryotic A-site, since it penetrates deeply into the major groove, thus displacing A1492 of 16S rRNA, giving the ribosome a continuous “on” state during the translation process. Structural studies of the interactions between aminoglycosides and the RNA or AMEs involved in their enzymatic inactivation have been performed to identify the molecular nature of these recognition processes. The 4,5 or 4,6-disubstituted aminoglycosides specifically bind to the A-site with several conserved contacts, the majority of them corresponding to the pseudo-disaccharide neamine [83,84,85]. The synthesis of hybrid (4,5/4,6-) aminoglycosides, based on the superimposition of crystallographic structures, originated a new family of 4,5,6-aminoglycoside derivatives with a better prognosis against AMEs than the corresponding natural antibiotics [86] (Figure 7). Another approach based on structural data showed that the antibiotic scaffold presents different conformations when bound to the A-site or to the AME. This realization translated into the design of a conformationally locked aminoglycoside that retained the antibiotic activity but was not susceptible to enzymatic modification [87,88] (Figure 7).

**Figure 8:**
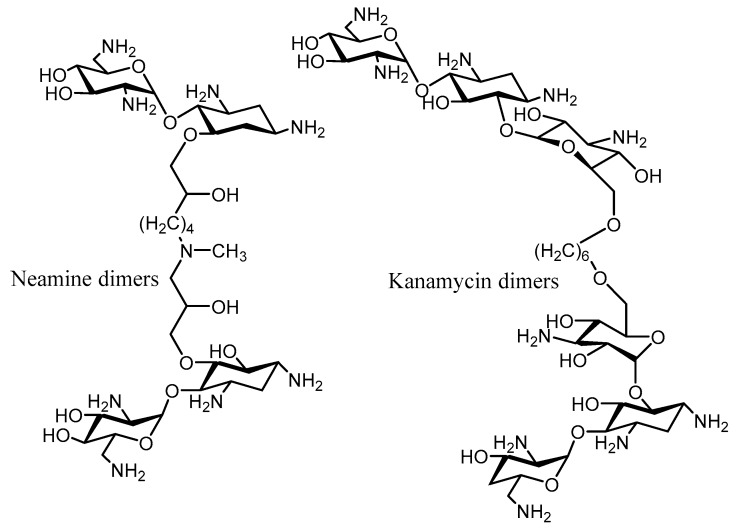
Structure of the Neamine and kanamycin dimers.

In order to increase the receptor binding affinity, aminoglycoside dimers were conceived and synthesized. Crystallographic studies indicated that neither the 6″-OH group in kanamycin nor the 5-OH group in neamine were essential for RNA binding; thus, they were selected as anchoring points for the synthesis of various dimers equipped with different linkers [89] (Figure 8). Kanamycin dimers (Figure 8) exhibited the same activity than the parent compound, but were also inactivated by the same AMEs (ANT(4′), APH(3′), and AAC(6′)). In the case of the neamine dimers [90] (Figure 8), these presented the same biological activity (lower than neomycin or kanamycin), but as well were inactivated by the AMEs. However, these compounds are interesting probes for strains not expressing AMEs, since they exhibit higher affinity for the receptor RNA.

The molecular recognition of aminoglycosides with their receptors, in most cases, is stabilized by a significant number of salt-bridges and polar contacts, but it seems to be promoted by CH/π stacking interactions involving aromatic residues of the protein/RNA with the antibiotic. So, the role played by CH/π stacking interactions in the molecular recognition of aminoglycosides by its receptors has been evaluated and proven to be significant [91]. The modification of natural aminoglycosides is a promising direction to search for novel aminoglycosides with potency against resistant strains.

**Figure 9:**
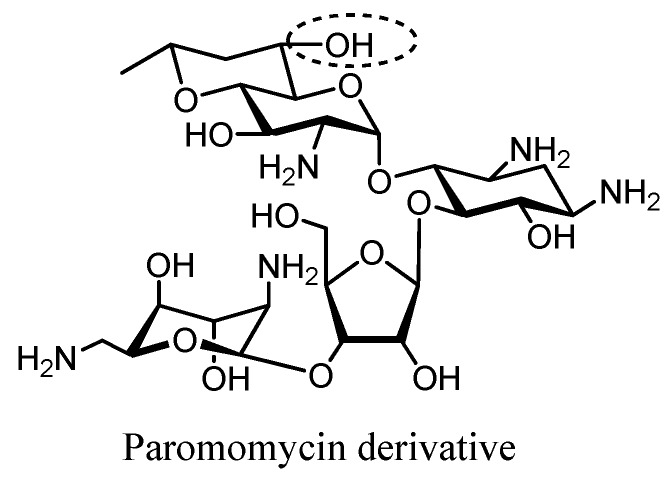
Structure of the Paromomycin derivative4. Inhibitors of Aminoglycoside Modifying Enzymes.

Recently, Crich et al. prepared a semisynthetic paromomycin derivative that displays similar antibacterial activity to the parent compound against clinical strains of *E. coli* and MRSA (ANT(4′,4″), APH-(3′,5″), and AAC(6′)). The enhanced activity on the ribosome has been demonstrated to depend on the equatorial hydroxyl group at the 6′-position, thereby providing support for the crystallographically derived models of aminoglycoside–ribosome interactions [92] (Figure 9).

**Figure 10:**
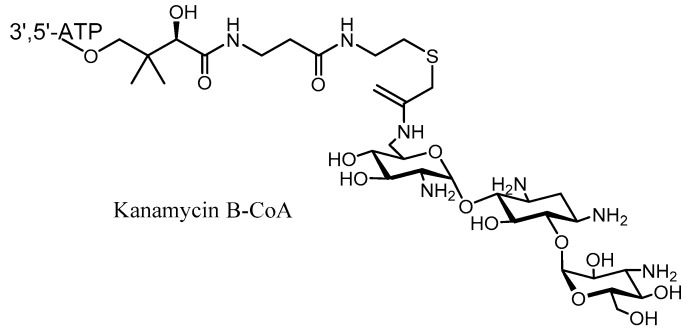
Structure of Kanamycin –CoA inhibitor to AME.

Another way to evade aminoglycoside resistance by AMEs is through the use of specific inhibitors of these enzymes. Some attempts have been made to produce inhibitors of one or more of the AMEs [93,94,95]. Bi-substrate analogs have been synthesized as inhibitors of AAC and ANT/APH enzymes based on the proposed kinetic mechanism. For instance, the crystallographic structure of AAC(6′)-Ii in complex with kanamycin B-CoA provided a new insight into chemical optimization [96] (Figure 10). This adduct is a good inhibitor of the AAC family, but unfortunately does not have any bacterial activity due to a poor permeability of the cell wall [97].

**Figure 11:**
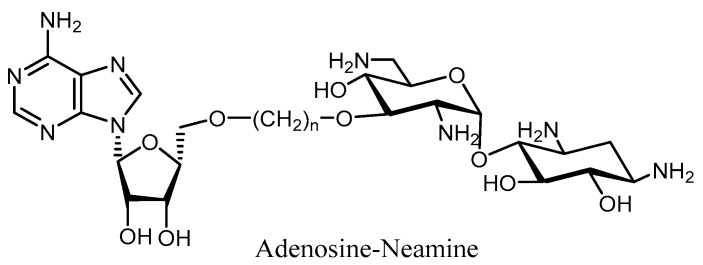
Structure of nucleotide-neamine complex as inhibitor of APHs and ANTs.

Using the same approach, a nucleotide–aminoglycoside complex for the inhibition of APHs/ANTs has been described (Figure 11). Such a tethered bi-substrate design contains a neamine core with the 3′-OH linked to adenosine via a non-hydrolyzable linker in place of the triphosphate group [98].

**Figure 12:**
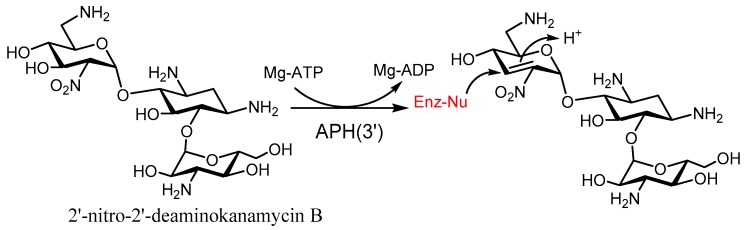
Structure and mode of action of the 2’-nitro-2’deaminokanamycin B.

Regarding the modification of discrete functional groups targeted by enzymatic resistance, different aminoglycoside analogs have been synthesized, which turn into suicide inactivators upon enzymatic phosphorylation. Such is the case of 2-nitro-2’-deaminokanamycin, a good inhibitor of APH(3′)-Ia and APH(3′)-IIa [99] (Figure 12), which generates in situ a nitro-alkene intermediate susceptible to Michael addition by close nucleophilic residues, thus rendering a covalent intermediate that blocks the active site and abolishes the activity (Figure 12). Another interesting example is the synthesis of 3′-ketokanamycin, where the keto group is known to exist in equilibrium with its ketal form, so that the phosphorylated ketal can be transformed back into a keto form by eliminating a dibasic phosphate, and then it can further re-regenerate the ketal. Interestingly, both 3′-ketal- and 2′-nitro-kanamycin derivatives can inactivate the APH(3′) in an irreversible manner. Most probably, these compounds are inhibitors of other kinases too.

**Figure 13:**
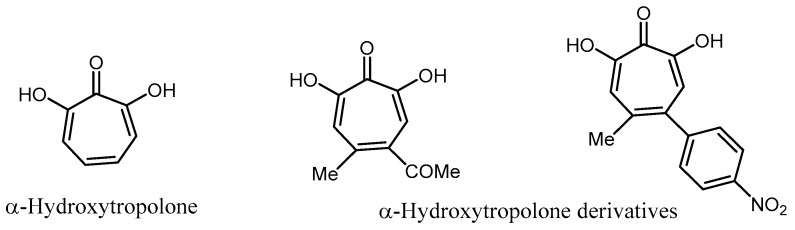
Structure of hydroxytropolone derivatives.

Allen et al. reported that α-hydroxytropolone plus the appropriate aminoglycoside substrates were active against resistant bacteria possessing the adenylyltransferase phenotype [100]. Recently, α-hydroxytropolone derivatives have also been described as good competitive inhibitors of ATP in the binding site of ANT(2″)-Ia [101] (Figure 13).

**Figure 14:**
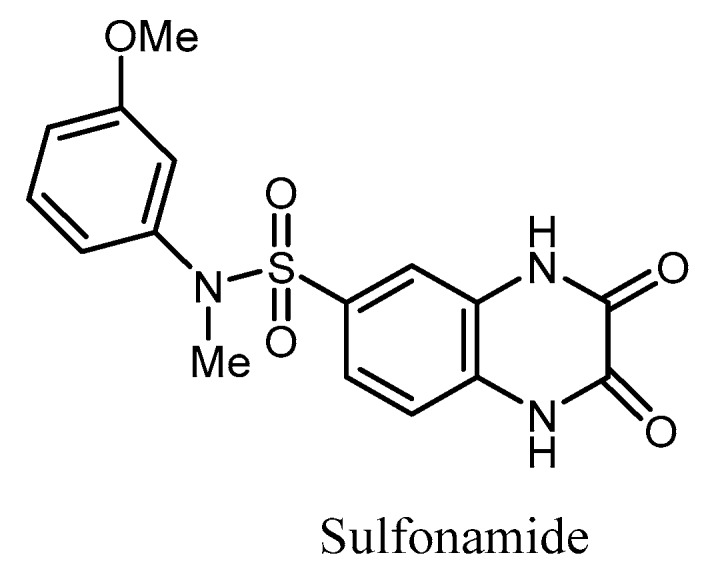
Structure of a sulfonamide as inhibitor of the AAC(2’’).

Garneau-Tsodikova et al. have reported a sulfonamide scaffold that served as a pharmacophore to generate inhibitors of AAC(2″) from *Mycobacterium tuberculosis*, whose upregulation causes resistance to the aminoglycosides [102] (Figure 14).

Given that AMEs share common binding features and that many of them also bind peptides and proteins, cationic peptides could serve as lead molecules in the development of new inhibitors of these enzymes. Therefore, cationic peptides have been tested as inhibitors of APH(3′)-IIIa, AAC(6′)-Ii, and AAC(6′)-APH(2″), and the results showed that the indolicidin moiety and its analogs have an inhibitory effect against both ACC and APH enzymes, albeit by different mechanisms. These peptides constitute the first example of broad-spectrum inhibitors of AMEs, but unfortunately none of them showed any inhibitory effect in vivo. Known inhibitors of eukaryotic protein kinases have been studied too to determine whether they were active against APH(3′)-IIIa and AAC(6′)-APH(2″) because of the structural relation found between these enzymes [95].

Some aminoglycoside dimers have also been used as inhibitors of the AMEs. Neamine dimers were investigated for their antibacterial activity and their capability to inhibit the action of bifunctional AAC(6′)-APH(2″), and were proven to be active against clinically isolated strains of *P. aeruginosa*. However, the synthesis of one universal inhibitor for all AMEs seems still unreachable, since good inhibition relies on many mechanistic and kinetic factors that can vary between families (AAC, ANT, and APH) and between types too (i.e., APH(3′)-I, II).

## 4. Combination Therapy

**Figure 15:**
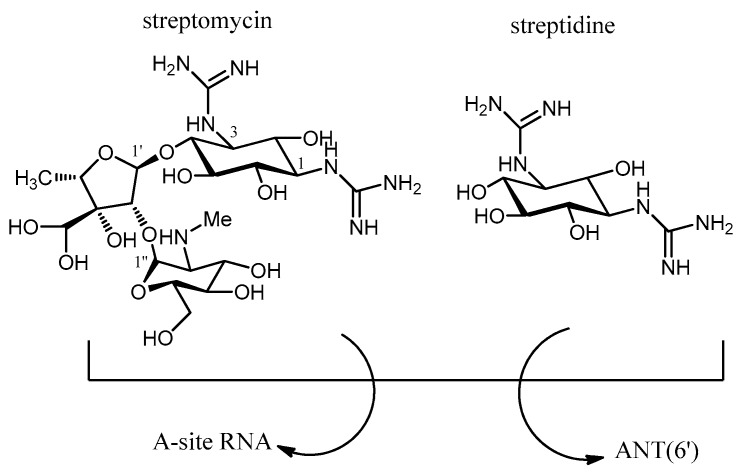
Structure of the streptomycin and streptidine as “decoy acceptor”.

The use of a combination therapy can help in solving the problem of resistance by AMEs. This approach relies on the rescue of original aminoglycosides (gentamicin, amikacin, or etimicin) through the co-administration of the corresponding inhibitors for each enzyme. Ideally, the adjuvant compound (inhibitor) would be targeted preferentially by the resistance enzymes, thus freeing the antibiotic to bind the target A-site. An all-purpose inhibitor would be a compound that mimics the charge and shape of an aminoglycoside (common for the three families of AMEs), having in mind that all AMEs have a highly negatively charged surface in their binding sites such that a must-have feature should be an overall positive charge. A proof-of-concept for this strategy is that the use of streptidine along with streptomycin in the cell culture restores the activity of the streptomycin against ANT(6), because streptidine competes for the binding site with the streptomycin, acting as a “decoy acceptor” of the enzyme [103] (Figure 15). Thus, streptidine could be a good starting compound for the design of more efficient “decoy acceptors” of AMEs targeting streptomycin, or 2-deoxystreptamine for those targeting 4,5- or 4,6-aminoglycosides (Figure 15).

**Figure 16:**
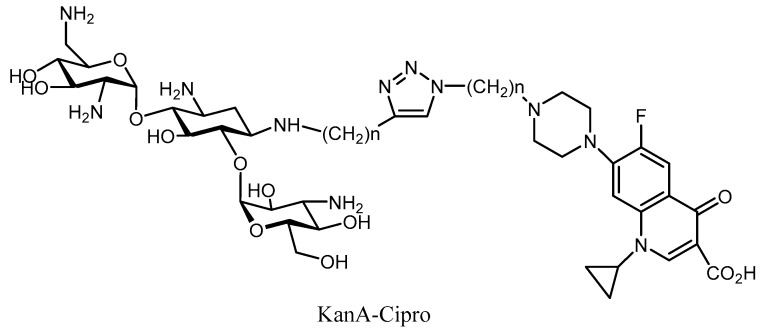
Structure of the kanamycin B-Cipro complex as hybrid antibiotic.

Hybrid antibiotics have also been developed to battle bacterial resistance [104,105,106,107]. The ability to slow down the emergence of resistance is probably one of the most important advantages of hybrid drugs [108,109]. This strategy connects two antibiotics that have different modes of action into a single molecule. The main difficulty so far has been to find the correct linker that connects the two drugs to provide better inhibition on both targets. The synthesis of the Cipro-NeoB/Cipro-KanA, which was active against a wide range of strains, is one of the most successful examples of this approach (Figure 16). The MIC values of these hybrids were the same against resistant and non-resistant strains (AAC(6′), APH(3′), and AAC(6′)/APH(2″) [110,111]. This kind of aminoglycoside derivative, based on a hybrid structure, provides a promising drug with an unusual dual mechanism of action, a potent profile against AMEs, and reduced potential for generating bacterial resistance.

## 5. Conclusions

In this review, we have aimed to cover the most relevant semi-synthetic aminoglycosides, inhibitors, and decoy acceptors of the AMEs. By far, the most successful chemical approach to modify the natural aminoglycosides has been the modification of the *N*-1/*N*-3″ amino groups with the AHB group or a guanidino substituent that has retained the parent antibiotic activity. Alkylation at the *N*-6′ position of the antibiotic resulted in a decrease of affinity for resistant enzymes, but a concomitant decrease in bacterial activity has been observed as well. Chemical deoxygenation of the 3′/3′,4′-OH groups of aminoglycosides resulted in compounds with high affinity for the RNA and resistance against MRSA. Comparatively, fewer inhibitors or decoy acceptors of the AMEs have been described.

On the other hand, ANT(4′)-Ia, ANT(2″)-Ia, ANT(3″)(9), AAC(3)-IIIb, and APH(3′)-IIIa have been described as highly dynamic enzymes and display properties of intrinsically disordered proteins in the absence of the aminoglycosides [112]. The degree of promiscuity by AMEs is governed by the dynamics of the protein, which is strongly influenced by the ligand and the cofactor, as well as its interaction with the solvent, and it should be taken into consideration in the design of new drugs.

Structural knowledge of both the RNA- and AME-aminoglycoside complexes has helped in the design of new antibiotics that can escape the action of the enzymatic modification [113]. In order to avoid or inhibit the activity of AMEs, obtaining structural and mechanistic information is of paramount importance.

## Figures and Tables

**Table 1:** *N*-acetyltransferase (AAC)-modifying enzymes.

Enzyme		Resistance Profile	Bacterial Source	Pdb Number
AAC(6′)	I (a–d,e,f–z)	T, A, N, D, S, K, I	*Salmonella enterica*	1S60, 2VBQ, 1S3Z, 1S5K, 2QIR
	II	T, G, N, D, S, K	*Enterococcus faecium*	2A4N, 5E96
			*Acinetobacter haemolyticus*	4F0Y, 4EVY, 4F0Y
			*Acinetobacter baumannii*	4E80
			*Escherichia coli*	6BFF, 6BFH, 1V0C, 2BUE, 2VQY
			*Staphylococcus warneri*	4QC6
AAC(3)	I (a–b)	G, S, F	*Serratia marcesans*	1B04
	II (a–c)	T, G, N, D, S	*Pseudomonas aeruginosa*	4YFJ
	III (a–c)	T, G, D, S, K, N, P, L	*Klebsiella pneumoniae,*	
	IV	T, S, N, D, S, A	*Campylobacter jejuni*	
	VII	G	*Actinomycetes*	
AAC(2′)	I (a–c)	T, S, N, D, Ne	*Providencia stuartii*	5US1
			*Mycobacterium tuberculosis*	1M44, 1M4D, 1M4G, 1M41
AAC(1)	Ia	P, L, R, AP	*E. coli*	
			*Campylobacter* spp.	

Abbreviations: A, amikacin; AP, apramycin; D, dibekacin; F, fortimicn; H, hygromycin; I, isepamicin; G, gentamicin; K, kanamycin; L, lividomycin; N, netilmicin; Ne, neomycin; P, paromomycin; R, ribostamycin; S, sisomicin; T, tobramycin.

**Table 2:** *O*-phosphotransferase (APH)-modifying enzymes.

Enzyme		Resistance Profile	Bacterial Source	Pdb
APH(3′)	I (a–d)	K, Ne, R, L, P	*Acinetobacter baumannii*	4FEV
	II	K, Ne, B, P, R	*Stenotrophomonas maltophilia*	
	III (a–b)	K, Ne, P, B, L, R, B, A, I		
	IV	K, Ne, B, P, R		
	V	Ne, P, R		
	VI	K, Ne, P, R, B, A, I	*Bacillus circulans*	
APH(2″)	I-a	K, G, T, S, D		
	I-(b,d)	K, G, T, N, D	*Escherichia coli*	4DCA
	II-(a–b)	K, G, T	*Enterococcus faecium*	3HAM, 3HAV
	IVa	G, K, S	*Enterococcus cassaliflavus*	5C4K, 5C4L, 4N57, 4DT8, 4DT9, 4DTA, 4DTB, 3SG8, 3SG9
APH(3″)	I (a–b)	St	*Acinetobacter baumannii*	4EJ7, 4FEU, 4FEV, 4FEX, 4FEW
	III a	St	*Enterococcus faecalis*	2BKK
APH(7)	I a	H	*Streptomyces hygroscopicus*	
APH(4)	I-(a–b)	H	*Escherichia coli*	3W0O, 3TYK, 3W0M, 3W0N
APH(6)	I-(a–d)	St	*Streptomyces griseus*	
APH(9)	I-(a–b)	Sp	*Legionella pneumophila*	3I0O, 3I0Q, 3I1A, 3Q2M

Abbreviations: A, amikacin; D, dibekacin; H, hygromycin; I, isepamicin; G, gentamicin; K, kanamycin; L, lividomycin; N, netilmicin; Ne, neomycin; P, paromomycin; R, ribostamycin; S, sisomicin; T, tobramycin; Sp, spectinomycin; St, streptomycin.

**Table 3:** *O*-nucleotidyltransferase (ANT)-modifying enzymes.

Enzyme	Resistance Profile	Bacterial Host	Pdb Number
ANT(2″)	K, T, G, D, S	*Pseudomonas aeruginosa*	4XJE, 5CFT, 5CFS, 5CFU
		*Klebsiella pneumoniae*	4WQK, 4WQL, 5KQJ
ANT(3″)	St, Sp	*Salmonella enterica*	4CS6, 5G4A
ANT(4′)	K, Ne, T, A, D, I	*Pseudomonas aeruginosa*	4EBJ, 4EBK
		*Staphylococcus aureus*	1KNY
ANT(6)	St	*Bacillus subtilis*	2PBE, 1B87
ANT(9)	Sp	*Enterococcus avium*	

Abbreviations: A, amikacin; D, dibekacin; I, isepamicin; G, gentamicin; K, kanamycin; Ne, neomycin; S, sisomicin; T, tobramycin; Sp, spectinomycin; St, streptomycin.

**Table 4:** Bifunctional modifying enzymes.

Enzyme	Resistance Profile	Bacterial Source	Pdb Number
		*Enterococcus faecalis*	
AAC(6′)-Ie-APH(2″)-IVa	G, K, T, A	*Staphylococcus aureus*	4ORQ
APH(2″)-Id-APH(2″)-IVa	K, G, T, S, D	*Enterococcus casseliflavus*	4DBX, 4DE4, 4DFB
APH(2″)-Ia-APH(6′)-Ie	K, G, T, S, D, St	*Staphylococcus aureus*	5IQF
ANT(3)-Ib-AAC(6′)-IId	T, A, N, D, S, K, St, Sp	*Serratia marcescens*	
AAC(3)-Ib-AAC(6′)-Ib	G, S, F, T, A, N, D, K, I	*Pseudomonas aeruginosa*	

Abbreviations: A, amikacin; D, dibekacin; F, fortimicn; I, isepamicin; G, gentamicin; K, kanamycin; N, netilmicin; S, sisomicin; T, tobramycin; Sp, spectinomycin; St, streptomycin.
